# Research Priorities to Expand Virtual Care Access for Patients in the Veterans Affairs Health Care System

**DOI:** 10.1007/s11606-023-08463-2

**Published:** 2024-01-22

**Authors:** Charlie M. Wray, Ursula Myers, Cindie Slightam, Navid Dardashti, Leonie Heyworth, Allison Lewinski, Peter Kaboli, Thomas Edes, Kevin Trueman, Donna M. Zulman

**Affiliations:** 1 Department of Medicine, University of California, San Francisco, San Francisco, CA USA; 2 Section of Hospital Medicine, San Francisco Veterans Affairs Medical Center, San Francisco, CA USA; 3 Charleston Health Equity and Rural Outreach Innovation Center (HEROIC), Ralph H. Johnson VA Medical Center, Charleston, SC USA; 4 Mental Health Service Line, Ralph H. Johnson VA Medical Center, Charleston, SC USA; 5 Department of Psychiatry and Behavioral Sciences, Military Sciences Division, Medical University of South Carolina, Charleston, SC USA; 6 VA Center for Innovation to Implementation (Ci2i), Menlo Park, CA USA; 7 Department of Veterans Affairs, New York Harbor, NY USA; 8 Department of Veterans Affairs Central Office, Office of Connected Care/Telehealth, Washington, DC USA; 9 Center of Innovation to Accelerate Discovery and Practice Transformation, Durham Veterans Affairs Health Care System, Durham, NC USA; 10 School of Nursing, Duke University School of Medicine, Durham, NC USA; 11 Veterans Rural Health Resource Center-Iowa City, VA Office of Rural Health, and Center for Access and Delivery Research and Evaluation (CADRE) at the Iowa City VA Healthcare System, Iowa City, IA USA; 12 Department of Internal Medicine, University of Iowa Carver College of Medicine, Iowa City, IA USA; 13 Office of Geriatrics & Extended Care, Department of Veterans Affairs, Washington, DC USA; 14 Department of Veterans Affairs, Independence, OH USA; 15 Division of Primary Care and Population Health, Stanford University School of Medicine, Stanford, CA USA

**Keywords:** telemedicine, virtual care, health services accessibility, Veterans Health Administration

## Abstract

The rapid expansion of virtual care is driving demand for equitable, high-quality access to technologies that are required to utilize these services. While the Department of Veterans Affairs (VA) is seen as a national leader in the implementation of telehealth, there remain gaps in evidence about the most promising strategies to expand access to virtual care. To address these gaps, in 2022, the VA’s Health Services Research and Development service and Office of Connected Care held a “state-of-the-art” (SOTA) conference to develop research priorities for advancing the science, clinical practice, and implementation of virtual care. One workgroup within the SOTA focused on access to virtual care and addressed three questions: (1) Based on the existing evidence about barriers that impede virtual care access in digitally vulnerable populations, what additional research is needed to understand these factors? (2) Based on the existing evidence about digital inclusion strategies, what additional research is needed to identify the most promising strategies? and (3) What additional research beyond barriers and strategies is needed to address disparities in virtual care access? Here, we report on the workgroup’s discussions and recommendations for future research to improve and optimize access to virtual care. Effective implementation of these recommendations will require collaboration among VA operational leadership, researchers, Human Factors Engineering experts and front-line clinicians as they develop, implement, and evaluate the spread of virtual care access strategies.

## BACKGROUND

As the largest integrated health care system in the USA, the Department of Veterans Affairs (VA) has long been a leader in the use of virtual care — herein defined as technologies intended to enhance the accessibility, capacity, quality, and experience of VA health care for Veterans, their families, and their caregivers, wherever they are located. Virtual care has known limitations including absence of physical exam data, possible overutilization of health care resources, and potential risk of exacerbating health and digital disparities due to inequitable access. Despite these limitations, prior research has also shown that virtual care is associated with improved patient outcomes and satisfaction,^1^ enhanced provider-patient communication,^2, 3^ reduction in the provider-patient supply-demand mismatch, and expedited access to specialist consultation.^4–6^ Given the needs of Veterans and these distinct advantages, the VA has heavily invested in virtual care programs in recent years.^7–9^ For instance, the VA currently funds over 20 virtual care programs through the Office of Rural Health,^10^ champions the use of its web-based patient portal system (MyHealth*e*Vet), utilizes synchronous and asynchronous telehealth modalities and remote patient monitoring, and supports over three dozen mobile apps that address a wide variety of medical conditions.^11^ As a result of these initiatives, the VA is currently the largest virtual care provider in the USA.^12^

Given the heterogeneous needs of VA’s patient population, maintaining consistent and equitable access to virtual care is an important priority. For instance, nearly one-quarter of all Veterans and one-third of Veterans who rely on VA for health care live in rural or highly rural areas,^13^ where virtual access to remote, specialized services could address care shortages. Additionally, the VA traditionally cares for individuals who are older, who may have cognitive or executive challenges (e.g., dementia), and who have more sociodemographic disadvantages (e.g., lower educational attainment, income, or social support) and less access to digital devices than other sectors of the population — further complicating virtual care access and potentially widening digital inequities.^14^ To address these challenges, policymakers and VA leadership have taken steps to enhance virtual care access, for example, by removing geographic licensure barriers to providing care across state lines in the MISSION Act,^15^ waiving copays for telehealth visits,^16^ and increasing training opportunities for VA providers.^17^ Although the COVID-19 pandemic spurred rapid implementation of virtual care across diverse clinical services, there is also a risk that the dramatic expansion since spring of 2020 could exacerbate the “digital divide” and worsen health inequities.^18, 19^ Recent studies have verified this concern, finding disproportionately lower use of video-based visits among VA patients who are older, those in rural areas, and those with history of homelessness.^20^

Given the rapid uptake of virtual care and the potential for digital inequities, the VA Health Services Research and Development Service (HSR&D) convened a state-of-the-art (SOTA) conference in May 2022 to develop research priorities for advancing the science, clinical practice, and implementation of virtual care. SOTA conferences aim to convene multidisciplinary groups of VA and non-VA experts to synthesize current evidence and identify gaps in the literature on topics critical to the health and well-being of Veterans, and to promote implementation of findings that improve quality of care.^21^ The conference focused on three primary domains: access, engagement, and outcomes in virtual care. Herein, we present the methods and consensus recommendations that emerged from the Access Workgroup at the 2022 VA Virtual Care SOTA conference.

## METHODS

In the 10 months preceding the SOTA conference in May 2022, the Access Workgroup Coordinating Committee (CMW, USM, CS, DMZ) met bi-monthly to refine key questions, identify subject matter experts, and select pre-conference reading materials. In total, 16 subject matter experts from disciplines including health services research, psychology, general internal medicine, geriatrics, nursing, human factors engineering, and health policy — as well as operations partners in virtual care, rural health, and health equity — participated in the Access Workgroup. Research leaders (CMW, UM) in the Access Workgroup conducted a rapid review to obtain recent publications on virtual care access, with a priority focus on VA populations to inform workgroup discussion. We searched PubMed, using MeSH terms for virtual care, telemedicine, and telehealth and limited the search to the prior 10 years to identify publications describing best practices and evidence for strategies that enhance access to virtual care. Literature review findings, summarized in Table 1, were provided to workgroup participants in advance of the conference along with three key questions to prepare for and focus the workgroup’s discussion about research priorities related to virtual care access. Those three key questions were as follows: (1) Based on the existing evidence about barriers that impede virtual care access in digitally vulnerable populations, what additional research is needed to understand these factors? (2) Based on the existing evidence about digital inclusion strategies, what additional research is needed to identify the most promising strategies? and (3) What additional research beyond barriers and strategies is needed to address disparities in virtual care access?

**Table 1:** Selected Findings from the Review of Literature Describing Virtual Care Access Barriers and Strategies

Barriers to virtual care access
Several sociodemographic characteristics common in the VA patient population have been shown to be associated with lower access and engagement with virtual care technologies.^20, 22–24^ These include: • Residing in rural areas • Older age • Identification as a racial/ethnic minority • Low socioeconomic status • Limited health literacy
Barriers to virtual care adoption stem from both the technology and operational issues^23, 25^: • **Technology-based barriers** (e.g., number of steps required to connect to a virtual visit, flexibility in using different types of video-capable platforms, and provision of free or low-cost infrastructure (including devices and internet access)) • **Operational barriers** (e.g., scheduling and staffing considerations, communication strategies between schedulers and providers, and provision of support or technical staff to assist patients on how to use virtual care technologies)
Some barriers to virtual care access are specific to types of clinical care • **Mental health**.^26^ Most reported barriers to video-based mental health care are related to clinical (e.g., can psychosis be assessed and managed appropriately during a video visit?), technical (e.g., is it feasible to troubleshoot connections?), and administrative (e.g., how do policies help or hinder providers?) concerns • **Primary care**.^7, 27^ Key implementation barriers in the VA include workforce training, patient education, and the need to expand the technology infrastructure • **Medical subspecialty care**.^7, 28^ Survey studies found subspecialty providers reported greater inabilities to conduct a physical exam or ability to assess physical health status than primary care providers
Strategies to improve virtual care access
The VA and the Office of Connected Care identified several strategies that facilitated virtual care expansion during the COVID-19 pandemic. Tactical areas that were critical to the VA’s success were^7^: • Training and support for the VA workforce^29^ • Expanding the technology infrastructure • Providing VA patients with technology (e.g., tablets) and/or internet service^30, 31^ • Focusing on high-risk patient populations^31, 32^
Other interventions that have the potential to improve virtual care access for Veterans^33–35^: • **Remove health system–created barriers** (e.g., offer video visits to every patient, offer telephone visits if unable to mitigate barriers to video visits, and increase system leadership awareness of barriers to telemedicine) • **Systematically assess individual patients’ access and digital literacy skills** (e.g., (1) develop protocols to assess patient technical readiness and needs at clinical intake and update at each subsequent visit, (2) ask patients what devices they own and how they access the internet, (3) incorporate this information into the EHR as a means of documenting virtual care access abilities, (4) track which individuals appear to be missing video visits, or relying on phone visits instead of video and develop outreach programs, (5) improve pre-visit preparation by providing instructions for connecting, conducting test visits to confirm capability) • **Provide classes or trainings to address digital literacy** (especially for high-risk groups identified above) • **Consider cultural and functional limitations** (e.g., ensure an option for language interpretation services and for hearing, vision, or cognitive impairment) • **Provide robust training for staff** (e.g., train clinical staff, peer support teams, volunteers, and/or health care navigation specialists to help patients manage EHR portals and telemedicine tools. Add telemedicine best practices to medical education and training curricula) • **Consider unique living situations and alternative access sites** (e.g., provide devices and a private space at group living sites, such as shelters, design and provide locations where individuals can join telemedicine visits that offer reliable connectivity, and privacy (e.g., accessible stations in a parking lot or library, or available devices and assistance at central community-based organization sites))

Following the plenary presentations on the first day of the SOTA Conference, members of the Access Workgroup convened for approximately 6 h of in-person discussion. First, the Workgroup identified virtual care access topics with sufficient evidence. Then, for each of the key questions, Workgroup members divided into groups of 4–5 individuals for discussion, and then reported out their respective discussion topics and research priorities to the larger group. All discussions were captured by note takers and shared on projected screen. Following these sessions, the Access Workgroup Coordinating Committee (CMW, UM, DZ, CS) synthesized research priorities emerging from all small- and large-group discussions. The full workgroup then convened, where in-depth discussions were held refine and finalize the list of research priorities. Consensus was achieved when all members verbally agreed on the final list of research priorities.

On the second day of the SOTA Conference, Access Workgroup coordinators presented a summary of their discussions and their consensus research priorities to all SOTA attendees (i.e., discussants and participants from the access, engagement, and outcomes workgroups). Following this presentation, an open discussion was held for further comments, suggestions, and clarifications, followed by responses from VA leadership in health services research and virtual care. Finally, the Access Workgroup Coordinating Committee consolidated all comments and finalized the research priorities. This final version was shared with all members of the Workgroup to ensure there was consensus, and all Workgroup members had an opportunity to comment, refine, or edit the material presented in this manuscript.

## RESULTS

### Established Evidence Regarding VA Virtual Care Access

Prior to the Access Workgroup’s discussion about research priorities, Workgroup members highlighted topics related to virtual care access *barriers* and *strategies* for which there is substantial evidence and therefore lower urgency for additional research. Within the *barriers* domain, the Workgroup noted that there was existing published literature describing the “digital divide” (i.e., the differential access to personal technology or broadband connectivity often based on social, economic, or demographic factors) and virtual care access patterns related to frequently studied sociodemographic characteristics (e.g., age, gender, race, rurality). Additionally, research has broadly shown that barriers to virtual care access exist within and across multiple levels within the health care system, including the patient, provider, and systems levels. Within the *strategies* domain, the Workgroup noted that virtual care expansion is feasible (as observed during the COVID-19 pandemic) and can be facilitated by providing Veterans with devices and Wi-Fi connectivity. In addition, substantial evidence indicates that changing providers’ behaviors and habits can be difficult and that virtual care training for both patients and providers needs to be tailored to one’s ability, comfort, interest, and setting. Finally, the Workgroup members described several virtual care access strategies with evidence suggesting promise, including the VA’s Digital Divide Consult that can be used to refer patients for video-enabled devices and internet service,^30^ and eConsults, which can be used to offer Veterans access to chart review and consultation from remote specialists.^36^ Many of these findings were highlighted in the review of published literature summarized in Table 1.

### VA Virtual Care Access Research Priorities

The following six recommendations emerged from the Access Workgroup’s discussions as priority research areas to better understand barriers and to identify strategies to increase VA virtual care access and digital inclusion. Examples of potential research questions within each priority area are presented in Table 2.

**Table 2:** Research Priorities and Examples of Specific Study Questions That Emerged from Discussions of the Access Workgroup at the 2022 Virtual Care State-of-the-Art Conference

Research priority	Potential research topics
Identify and evaluate opportunities to optimize Veterans’ access to virtual care interventions at the patient, provider, and systems level	*Patient level* • Are there specific training/teachings that can enhance Veteran’s knowledge, skills, and interest in virtual care? • What type of technical and decision support is needed to improve Veteran’s virtual care access (e.g., Virtual Health Resource Center)? • How do we capitalize on specific clinical events (e.g., an Emergency Department visit or hospitalization) to support a Veteran in gaining access to virtual care?
	*Provider level* • What interventions work best to train providers to optimally utilize virtual care? • What are the most effective strategies to disseminate best practices? • What is the impact of provider-level incentives on virtual care use?
	*Systems level* • What structural changes and/or policies are needed to optimize a Veteran’s access to virtual care? • What are specific implementation strategies that can optimize the reach and penetration of virtual care interventions? • How can the VA foster a culture that supports widespread virtual care access?
Create standardized virtual care access metrics with the goal of tracking access expansion and equity	• How can virtual care access metrics help in measuring changes in the digital divide? • How can metrics be used to measure the effectiveness of virtual care access interventions? • Are there quantitative measures of a patient’s virtual access, capability, and/or use that can be used to assess the impact of virtual care on quality and health outcomes?
Customize technology, implementation strategies, and virtual care models to ensure equitable virtual care access	• How could the expansion of virtual care access exacerbate certain disparities?
Examine how the VA can offer access to virtual care that meets a veterans’ dynamic clinical needs and social circumstances	• What is the optimal modality and combination of virtual care and non-virtual care for a given patient given their social, clinical, and economic circumstances? • How does the VA identify and prevent excess, ineffective or inappropriate virtual care access? • How can virtual care encourage Veterans to choose VA care over community care?
Identify implementation strategies that increase patients and clinician adoption of effective virtual care technologies	• How can variations in virtual care access across clinics, facilities, and VISNs offer opportunities to learn from positive outliers and scale those successful interventions rapidly? • How can the VA leverage implementation science to enhance virtual care access (e.g., increase awareness of new and existing virtual care services, interventions, and resources)?
Identify rapid, real-time evaluation methods to optimize virtual care access	• How can the VA leverage its informatics infrastructure to better evaluate virtual care access? • What specific methods can be used to provide rapid, iterative design and evaluations of virtual care interventions?

1. Identify and evaluate opportunities to optimize Veterans’ access to virtual care interventions at the patient, provider, and systems level

Within this specific priority area, there exists three levels — patient, provider, and system — where more virtual care access research is needed. Because of the cross-cutting needs for research within this priority area, the Workgroup chose to describe research opportunities within each of these levels.

*Patient level*. Opportunities for access-related research focused on patient needs and experiences include, but are not limited to, studies within the following domains: patient trust (e.g., understanding how a Veteran’s virtual care access is influenced by their trust in the health care system), individual preference (e.g., acknowledging individuals’ personal and circumstantial preferences for either virtual or in-person care), access to devices and Wi-Fi, and digital literacy.^20, 22, 37–39^ While the COVID-19 pandemic accelerated research in many of these areas, further studies are needed to explore which of these domains are modifiable, determine the highest impact and most efficacious strategies to increase an individual’s access to virtual care, and identify interventions and strategies that strengthen Veterans’ digital literacy.

*Provider level*. Providers play a major role in facilitating and encouraging virtual care access. Research is needed to more fully understand how providers’ behaviors are shaped by their own virtual care knowledge, skills and abilities, comfort, and confidence, as well as their underlying perceptions about the value and quality of virtual care. Although recent literature highlights broad support among VA providers for the continued use of virtual care,^28^ more work is needed to explore how virtual care use impacts a provider’s clinical workload, job satisfaction, workflow, and work-life balance — and how these factors influence sustained use of virtual care. Future research should also explore providers’ potential biases about patients’ interest in and capacity to use virtual care, their perception of virtual care quality and its abilities to address their clinical needs, and their knowledge about patients’ virtual care preferences and capabilities.

*Systems level*. Systems-level research is needed to understand how virtual care access is shaped by culture (at the facility and community level), climate (e.g., structures, processes, equipment, ease of use and policies), and workforce (e.g., distribution across geographic areas and the presence or availability of specialized providers). Recent studies have found that virtual care adoption was not uniform across the VA and that certain facility-level characteristics were often associated with more or less virtual care uptake (e.g., facilities with poor broadband coverage and less prior virtual care experience).^23^ Additional research is needed to elucidate strategies that target these specific factors.

2. Create standardized virtual care access metrics with the goal of tracking access expansion and equity

Because virtual care represents a paradigm shift for health service delivery, both within and outside the VA system, standardized virtual care access metrics are needed to evaluate reach and equity of access interventions. Validated measures will facilitate rigorous, reliable, and comprehensive tracking of virtual care access expansion and, importantly, help ensure equitable access to virtual care across the entire VA enterprise. The VA has implemented a number of standardized access metrics nationwide^40, 41^ (e.g., new and established patient wait times, third next available appointment times, and same-day access) and is well-positioned to lead research and implementation efforts that will be informative for other systems.

3. Examine how the VA can offer access to virtual care that meets a Veterans’ dynamic clinical needs and social circumstances

Veterans’ clinical, social, and economic needs ebb and flow based on numerous factors in their lives. Noting this, future research should consider the dynamic nature of each Veteran’s life and experience, and factor in their clinical trajectory, changing social risk, and evolving digital literacy over time. One opportunity is to identify the optimal virtual/non-virtual care modalities for a given person based on their clinical and social needs at that moment — thereby offering individuals the “right care in the right place at the right time.”^42^ Concurrently, as virtual care becomes more standard and widespread, studies should consider the potential for excess and/or inappropriate virtual care, how to universally improve digital literacy skills among at-risk Veterans, and where there may be a need for de-implementation. As a Veteran’s capabilities, clinical needs, and social risks are dynamic, research is needed to ascertain when virtual care is likely to be adequate and when it is not. Finally, as a Veteran’s needs change over time, research should examine whether virtual care may offer flexibility in supporting Veterans to choose VA care over community care, when appropriate.

4. Identify implementation strategies and approaches that increase patient and clinician adoption of effective virtual care technologies

Given the rapid need to understand how to equip Veterans and providers with the tools and skills to access virtual care, future research should help identify implementation barriers and facilitators and their relationship with virtual care access patterns. As a Learning Health Care System that aims to integrate clinical informatics, incentives, and culture to promote continuous improvement and innovation, the VA is well-positioned to achieve this goal.^43^ For example, there may be opportunities to study high-performing VA clinics or facilities in order to identify and disseminate effective implementation strategies. Leveraging implementation and delivery science methodologies and approaches (e.g., hybrid studies and stepped-wedge trials) will be a key feature in improving access to virtual care technologies within the VA.

5. Identify rapid, real-time evaluation methods to optimize virtual care access

While ensuring access to high-quality virtual care is paramount, there is also a need for rapid, rigorous evaluation given the speed with which the digital landscape evolves. Research should consider how best to (1) incorporate user-centered design methods to improve virtual care accessibility, (2) integrate informatics approaches (e.g., usability testing, dashboards) to inform best practices, and (3) leverage big data and machine learning approaches to identify patient populations with access needs. The VA has a long and developed history in the use of implementation science and other community-engaged research methods to promote the uptake of research into clinical practice.^44^ Virtual care researchers should continue to build on these methods and leverage the developed methodologic expertise located within the VA enterprise to optimize virtual care access.

6. Customize technology, implementation strategies, and virtual care models to ensure equitable virtual care access

Although virtual care has strong potential to address access disparities and service delivery barriers, the rapid uptake and use of technology remain a threat to equitable access to care and could potentially worsen the “digital divide.” The workgroup discussed the need to make equity a cross-cutting priority, such that equity principles and measures are integrated into all virtual care research and should be applied to each of the priority areas mentioned here. To facilitate this imperative, future research should further define and describe specific barriers contributing to inequities in virtual care access. Studies should consider special populations (e.g., older adults, those with complex medical needs, those who live in rural locales, and the socioeconomically disadvantaged) that may benefit from targeted intervention and outreach and tailored implementation strategies. Additionally, future research should examine potential equity-related adverse consequences that could arise from virtual care interventions and policy. For example, if virtual care is proven to be highly effective and disproportionately caters to younger populations, those who are more digitally vulnerable because they do not possess the necessary technology and digital literacy to use virtual care may be at risk for adverse outcomes.^45^ On the other hand, interventions intending to improve access should ensure that vulnerable populations are not induced into using virtual care products or systems with unclear effectiveness.^46^

## LIMITATIONS

The production of these recommendations may have limitations that should be considered. First, our workgroup did not establish priority levels for each of these recommendations, nor did we discuss the logistics, funding, and support needed to accomplish them. Second, while our workgroup did use research outside of the VA to help guide discussions, workgroup members were all affiliated with the VA, and our final recommendations are aimed at improving access to virtual care within the VA health care system. Third, while we did have a Veteran representative in the Workgroup, their experiences with digital and virtual based care may not reflect all Veterans’ experiences. Finally, while workgroup members included a broad range of experts who could comment and contribute to the discussions, some stakeholders’ voices and concerns may not be fully reflected in these recommendations.

## CONCLUSIONS

Virtual care will remain an important tool as the VA continues to maintain consistent access to care for all Veterans. The virtual care access workgroup highlighted research initiatives that health services researchers, clinicians, and VA policymakers should consider as more work is performed in this area. Moving forward, interdisciplinary collaboration across research groups and VA operations offices (e.g., Offices of Connected Care, Rural Health, Primary Care, Geriatrics, and other clinical services) can help to maximize resources and advance virtual care access issues.
